# Taking health systems research to the district level: a new approach to accelerate progress in global health

**DOI:** 10.1186/1472-6963-13-S2-S11

**Published:** 2013-05-31

**Authors:** Agnes Binagwaho, Cameron T Nutt, Parfait Uwaliraye, Claire M Wagner, Jean Pierre Nyemazi

**Affiliations:** 1 Ministry of Health of Rwanda, Kigali, Rwanda; 2 Harvard Medical School, Boston, USA; 3 Geisel School of Medicine, Dartmouth College, Hanover, USA; 4 The Dartmouth Center for Health Care Delivery Science, Dartmouth College, Hanover, NH, USA; 5 Global Health Delivery Partnership, Boston, USA; 6 Rwanda Biomedical Center, Kigali, Rwanda

## Introduction

The world recently marked the tenth anniversaries of the launch of both The Global Fund to Fight AIDS, Tuberculosis, and Malaria and the United States President’s Emergency Plan for AIDS Relief (PEPFAR). A decade ago, few could have possibly imagined how different the world would look today from the future we feared — a future where the country where one lived determined *if* one lived and where the rich increasingly tried to seal themselves off from the poor. Due to the courage of tens of thousands of activists across the globe who put their bodies on the line, the innovation of scientists who refused to relent in the face of complexity, and the vision of policymakers across six continents who imagined a more just and healthy future, we live today in a world that has started to be defined more by our connections than our differences.

This is global health: We have come a long way, but the work is not finished. As the world learned through the challenge of scaling up access to prevention, care, and treatment for HIV, tuberculosis, and malaria in countries with sparse health infrastructure, simply having the tools is not enough. Bypassing the public sector — weak as it might be at the outset — to get pills to patients faster may have been easier and did address some of the symptoms of decades of nonexistent access to quality health care. But this approach could never guarantee the poorest and most vulnerable access to health as a *right*[1]. That, it is now clear, requires a financially and geographically accessible health system complete with robust supply chains, an adequately staffed workforce with the right skill level and skill mix, and feedback loops driven by information and research — all rooted in the foundation of accountability provided by a strong and transparent public sector is required [2].

## Next steps, new approaches

**Figure 1:**
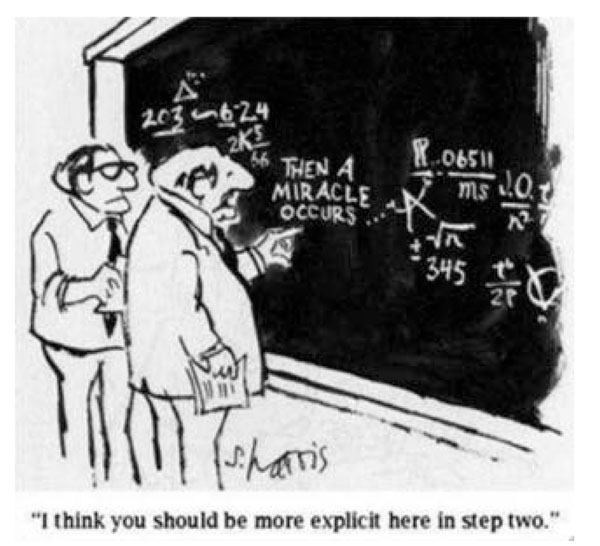
**A place for implementation research in global health.** ©Sidney Harris with permission [http://sciencecartoonsplus.com]

The collection of papers in this special issue of *BMC Health Services Research*[3] tackles some of the highest-priority questions for attaining and sustaining the benchmarks of a health system with sufficient breadth and depth to meet the needs of a population across the life course. International and interdisciplinary teams working in five countries (Ghana, Mozambique, Rwanda, Tanzania, and Zambia) present their approaches to applying the discipline of science to research on the complex challenges of service delivery. Narrowing the gulf between what we know and what we actually do about the leading causes of premature mortality and disability requires nothing less [4]. Whatever terminology one prefers — whether health services research, implementation science, or global health delivery — the commitment to rigorous scientific study of health care delivery is the next frontier for all who seek to advance the human right to health **(**Figure 1**)**[5,6].

Perhaps the most novel aspect of this collection is not simply its subject material or the rich diversity of approaches and contexts it includes, but rather the fact that these study designs and early lessons are being disseminated for scientific debate and critical analysis well ahead of traditional research timelines. Moreover, these research partnerships and their sponsor, the African Health Initiative at the Doris Duke Charitable Foundation, are committed to learning about and sharing not only successes, but also failures. Dissonance between expectation and result in global health has much to teach us about delivery, and the importance of widely sharing the valuable lessons that result cannot be understated [7].

Each of the research models included in this issue recognizes the value of an integrated approach to service delivery that is at once community-based, locally created and owned, and structured in a way conducive to constant learning. All of these models have been designed and launched by truly collaborative consortiums engaging ministries of health, academia, and non-governmental organizations to facilitate what might be termed a virtuous cycle of service provision, scientific research, and capacity building — all from the level of the district to the community, where the challenges of delivery are most urgent around the world.

## A view from Rwanda

The value of this approach has been acknowledged by many health workers in Rwanda, a country of 11 million that is now on track for the health-related Millennium Development Goals after coming from a situation where the health infrastructure and workforce were almost completely destroyed [8]. In the wake of decades of injustice that culminated in the 1994 genocide, the country’s largest priority was promoting social equity and ensuring that those rendered most vulnerable by the policies and the violence of the past had their needs prioritized. This meant a strong and rurally oriented health system. While funds were scarce at first, the advent of global solidarity for health in the early 2000s provided an avenue for health systems strengthening by beginning with the prevailing challenges of HIV, tuberculosis, and malaria.

It readily became apparent that the deadliest gaps in service delivery were not limited to individual pathologies, however; the same mother seeking to prevent transmission of HIV to her unborn child will also require antenatal care and a safe place to give birth, and a health worker to vaccinate her baby, as well as rapid treatment and referral should either of them fall sick with pneumonia. The Rwandan Ministry of Health and its partners, therefore, put new funds towards the development of comprehensive continuums of care linking the patient’s home to local health centers, district hospitals, and national referral facilities. By necessity and by design, Rwanda’s 45,000 community health workers constitute the backbone of the health system; recent experience has shown them to be essential not only for the reduction of child mortality, but also for the sustainable provision of high-quality care for a wide range of chronic conditions from HIV [9,10] and tuberculosis [11] to cardiovascular disease [12] and some cancers [13].

Driving policy decisions about the architecture and evolution of Rwanda’s health system have been mechanisms for collecting and strategically evaluating evidence in collaboration with partners from other sectors of government, academia, and international organizations. While it was clear that user fees were undermining access to care and causing catastrophic health expenditures among those who could least afford them, global prescriptions for health financing seemed poorly matched to Rwanda’s historical and social context at the turn of the millennium. Rwanda instead piloted a community-based health insurance scheme, *mutuelles de santé*, in three of the country’s 30 districts beginning in 1999 [14], which has since been scaled up to cover the entire country and rendered more progressive through the removal of all payments for the poorest quarter of the population following the results of subsequent evaluations [15]. A performance-based financing mechanism was also piloted via phased implementation starting in three districts in 2002 [16] and has been systematically evaluated and tweaked since [17]. These and other research efforts have catalyzed the health sector’s drive to ensure that disease-specific funding opportunities are used to strengthen the entire health system.

The PHIT Partnerships described in this issue by Peter Drobac and others [18] represent the next step in Rwanda’s efforts to link science with policy. We welcome the opportunity to analyze results from the other four countries and explore transferable lessons. As Rwanda and other countries around the world aim to incorporate the prevention, care, and control of non-communicable diseases into the health system and to build on past progress against infectious diseases [19], policymakers are looking for new approaches to bridge the global health implementation gap. The research designs and early lessons presented here may just be what is needed to fill it.

## List of abbreviation: used

PEPFAR: President’s Emergency Plan for AIDS Relief; PHIT: Population Health Implementation and Training

## Competing interests

AB is a co-investigator for the Rwanda Population Health Implementation and Training Partnership that is funded by the Doris Duke Charitable Foundation, though does not receive any financial compensation for her participation in the research.

## Authors’ contributions

AB conceived the argument. CTN wrote the first draft of the manuscript. AB, CTN, PU, CMW, and JPN contributed to the literature review and revised the manuscript critically for content.

## Authors’ information

AB is Minister of Health of Rwanda, Senior Lecturer at Harvard Medical School, and Clinical Professor of Pediatrics at the Geisel School of Medicine at Dartmouth. CTN is a research fellow with the Dartmouth Center for Health Care Delivery Science. PU is Director General of Planning, Monitoring and Evaluation, and Health Information Systems in the Ministry of Health of Rwanda. CMW is a research fellow with the Global Health Delivery Partnership. JPN is Director of Planning, Monitoring, and Evaluation in the Rwanda Biomedical Center in the Ministry of Health, and is a student in Dartmouth’s Master of Health Care Delivery Science program.

## Declarations

This article has been published as part of *BMC Health Services Research* Volume 13 Supplement 2, 2013: Improving primary health care to achieve population impact: the African Health Initiative. The full contents of the supplement are available online at http://www.biomedcentral.com/bmchealthservres/supplements/13/S2. Publication of this supplement was supported by the African Health Initiative of the Doris Duke Charitable Foundation.
